# CAR-T细胞桥接异基因造血干细胞移植治疗弥漫大B细胞型Richter综合征1例并文献复习

**DOI:** 10.3760/cma.j.cn121090-20250830-00406

**Published:** 2026-02

**Authors:** 阳 王, 娜 高, 增艳 刘, 媛媛 董, 晓萌 秦, 晓 刘

**Affiliations:** 滨州医学院附属医院血液科，滨州 256603 Department of Hematology, Binzhou Medical University Hospital, Binzhou 256603, China

## Abstract

本研究旨在探索嵌合抗原受体T细胞（CAR-T细胞）治疗桥接异基因造血干细胞移植（allo-HSCT）在弥漫大B细胞型Richter综合征患者中的应用，并综述该疾病的诊断、发病机制及治疗进展。本文回顾性分析滨州医学院附属医院1例确诊Richter综合征患者的诊疗过程并进行文献复习。患者为49岁女性，有慢性淋巴细胞白血病（CLL）病史2年余，因颈部右侧包块进行性肿大就诊，最终确诊为CLL转化为弥漫大B细胞淋巴瘤（DLBCL），即Richter综合征。患者先后接受3个周期R-DA-EPOCH联合泽布替尼方案治疗取得部分缓解，之后病情出现进展，调整方案为抗PD-1单抗、抗CD20单抗、XPO1抑制剂及泽布替尼联合治疗1个周期，继而接受自体来源的抗CD19 CAR-T细胞输注并桥接allo-HSCT。患者移植后3个月及8个月评估均显示疾病完全缓解。

## Abstract (en)

This study aimed to explore the application of CAR-T cell therapy bridging to allogeneic hematopoietic stem cell transplantation（allo-HSCT）in patients with diffuse large B-cell Richter syndrome, and to review recent advances in the diagnosis, pathogenesis, and treatment of this disease. We retrospectively analyzed the diagnosis and treatment course of one patient diagnosed with Richter syndrome at Binzhou Medical University Hospital and reviewed relevant literature. The patient was a 49-year-old female with a history of chronic lymphocytic leukemia（CLL）for over two years, who presented with a progressively enlarging right neck mass, and was ultimately diagnosed with transformation of CLL into diffuse large B-cell lymphoma（DLBCL）, i.e., Richter syndrome. The patient initially achieved a partial response after three cycles of R-DA-EPOCH combined with zanubrutinib therapy. Following disease progression, the treatment regimen was adjusted to a combination of PD-1 monoclonal antibody, CD20 monoclonal antibody, XPO1 inhibitor, and zanubrutinib for one cycle. This was followed by infusion of autologous anti-CD19 CAR-T cells and subsequent bridging to allo-HSCT. Assessments at 3 and 8 months post-transplantation both demonstrated disease complete remission.

Richter综合征（Richter syndrome，RS）指慢性淋巴细胞白血病（CLL）或小淋巴细胞淋巴瘤（SLL）向恶性程度更高的侵袭性淋巴瘤进展后，所表现出的一系列临床症候群和疾病状态[1]。CLL/SLL患者发生转化时，最常见的类型即为弥漫大B细胞淋巴瘤（DLBCL）[2]。一旦患者诊断RS，往往提示预后较差，中位生存期仅为5～8个月[3]。RS发病隐匿，全球范围内尚未形成统一的治疗标准。本文全面分析1例从CLL向DLBCL转化的RS患者的治疗过程，探讨化疗后采用异基因造血干细胞移植（allo-HSCT）、嵌合抗原受体T细胞（CAR-T细胞）治疗等策略治疗的可能，以期为临床实践提供参考。

## 病例资料

患者，女，49岁，因“确诊慢性淋巴细胞白血病2年余，颈部肿物进行性增大半年”于2024年5月28日入我院。患者约2年前因“白细胞增高”就诊当地医院，查体可触及颈部双侧、双侧腋窝、左侧腹股沟多发肿大淋巴结。完善浅表淋巴结及肝脾超声可见，颈部较大淋巴结为2.5 cm×1.4 cm；腋窝较大淋巴结为3.9 cm×1.5 cm；左侧腹股沟较大淋巴结为0.7 cm×0.4 cm；肝脏大小形态正常；脾稍大，长径约12.0 cm、厚约4.2 cm。完善骨髓穿刺等相关辅助检查后诊断为CLL。结合病史及相关辅助检查结果，患者未达到治疗指征，定期随访观察。半年前颈部右侧出现肿物（直径约8.0 cm），伴胀痛、乏力。既往高血压病史。入院查体：颈部双侧可触及多枚质硬肿大淋巴结，较大者约5.0 cm×4.0 cm，活动性差且胀痛明显；心、肺、腹部查体未见明显异常。血常规：WBC 38.4×10^9^/L，RBC 4.6×10^12^/L，HGB 118 g/L，PLT 170×10^9^/L，淋巴细胞百分比90.4％，中性粒细胞百分比7.3％，单核细胞百分比2.0％，嗜酸性粒细胞百分比0.1％，嗜碱性粒细胞百分比0.2％。生化：乳酸脱氢酶611.5 U/L，α羟丁酸脱氢酶412.2 U/L；β_2_微球蛋白2.83 mg/L。骨髓检查，形态学符合CLL（图1A）；流式细胞术检测到85.81％的CLL肿瘤细胞，病理证实为CLL/SLL（淋巴细胞占比为50％，免疫表型为CD20^+^/CD5^+^/CD23^+^）；染色体核型为46,XX[20]。颈部右侧淋巴结活检确诊为DLBCL，非生发中心亚型。免疫组化：CD20、PAX5、CD43、BCL2、MUM-1表达阳性，c-Myc约40％表达阳性，CD3、CD5、CD23、cyclinD1、CD10、BCL6表达阴性，Ki-67阳性率约80％（图1B～H）。FISH检测示P53基因（17p13.1）缺失。免疫球蛋白重链可变区（IGHV）测序证实与CLL为同一克隆起源。基线PET-CT示颈部右侧融合淋巴结（9.4 cm×7.4 cm，SUVmax 30.0）伴多区域淋巴结及骨骼^18^F-氟代脱氧葡萄糖（FDG）代谢增高。

**图1:**
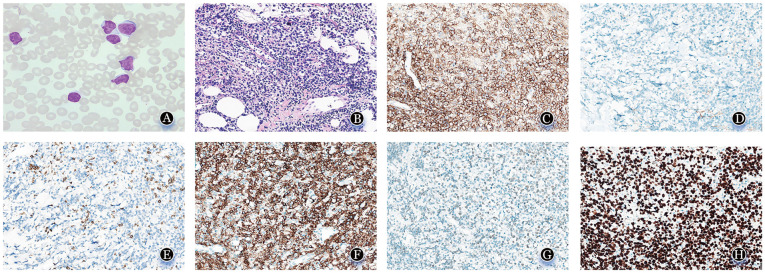
弥漫大B细胞型Richter综合征患者骨髓形态及颈部右侧淋巴结常规病理、免疫组织化学染色（×100） **A** 骨髓增生活跃，粒红巨三系增生骨髓象，细胞形态未见明显异常（×100）；**B** 可见中等大小淋巴样细胞弥漫分布并浸润周围脂肪组织（HE染色，×40）；**C** CD20染色：肿瘤细胞CD20弥漫阳性，阳性信号位于细胞膜；**D** 肿瘤细胞CD10阴性；**E** 肿瘤细胞CD5阴性；**F** 肿瘤细胞BCL2弥漫阳性，阳性信号位于细胞膜；**G** 肿瘤细胞BCL6阴性；**H** Ki-67增殖指数约80％

2024年6月5日患者开始接受R-DA-EPOCH（利妥昔单抗、依托泊苷、泼尼松、长春新碱、环磷酰胺、多柔比星脂质体）联合布鲁顿酪氨酸激酶抑制剂（BTKi，泽布替尼）方案治疗3个周期。8月5日经PET-CT评估显示部分缓解（Deauville评分4分，SUVmax 5.5）。8月9日患者接受原方案巩固治疗1个周期。9月1日患者颈部左侧出现新发肿块，穿刺病理示淋巴组织反应性增生（Ki-67阳性率为2％，CD20弱阳性）。结合病史及穿刺病理局限性，不排除淋巴结肿大与血液原发病相关。遂调整治疗方案，给予抗PD-1单抗（替雷利珠单抗）+抗CD20单抗（泽贝妥单抗）+XPO1抑制剂（塞利尼索）+泽布替尼联合治疗1个周期。患者于9月5日采集淋巴细胞制备抗CD19 CAR-T细胞（由山东省脐血库提供），10月6日接受氟达拉滨、环磷酰胺（FC）方案清淋预处理，10月9日回输抗CD19 CAR-T细胞（12.58×10^7^个），输注后第11天检测CAR-T细胞扩增峰值（61.28％）。11月19日复查PET-CT示进一步缓解（Deauville评分2分，SUVmax 1.9）。患者于11月30日接受allo-HSCT，预处理方案为塞替派/美法仑/环磷酰胺联合抗T淋巴细胞球蛋白。环孢素A、短程甲氨蝶呤、吗替麦考酚酯联合应用预防移植物抗宿主病（GVHD）。并于12月6日、7日回输供者（患者女儿，A+供A+，HLA 6/12相合）外周血干细胞（CD34^+^细胞共2.98×10^6^个/kg）。移植后第10天（+10 d）中性粒细胞植活，+14 d血小板植活。移植后2个月，患者出现腹泻、血压增高。完善相关检查示血细胞进行性下降，肌酐、乳酸脱氢酶水平进行性增高，补体C5b9水平增高（325 ng/ml），ADAMTS13活性正常（87.49％）。外周血涂片镜检可见破碎红细胞。肠镜未见明显异常。考虑合并移植相关血栓性微血管病（TA-TMA），加用甲泼尼龙、吗替麦考酚酯、抗CD25单抗，辅以血浆置换、促造血及成分输血及其他支持治疗，患者病情好转。2025年3月17日复查PET-CT示持续缓解（Deauville评分2分，SUVmax 1.8），骨髓形态学、流式细胞术检测无异常，供者嵌合度100％。2025年8月12日再次复查骨髓形态学、骨髓流式细胞术检测无异常，供者仍为嵌合度100％。截至2025年8月20日，患者一般状态良好，未出现GVHD。

## 讨论及文献复习

RS是CLL的一种严重并发症，本文报道1例CAR-T细胞治疗桥接allo-HSCT治疗弥漫大B细胞型RS患者的临床案例，以期提升临床医师对RS的认识。1928年Richter首次报道1例CLL患者短期内死亡，尸检见多发淋巴结及肝脾肿大，对异常病变行病理分析，发现同时存在CLL细胞和“多形性组织细胞”[4]，后证实为转化的大B细胞[5]。CLL患者出现淋巴结快速增大、发热、盗汗、不明原因体重下降等表现时均应考虑RS，乳酸脱氢酶升高及高钙血症亦有提示意义。影像学上，PET-CT可协助RS诊断，其有助于定位可疑病灶以引导活检（优先推荐淋巴结切除或粗针穿刺），但诊断SUVmax阈值（如≥5或≥10）的敏感性和特异性仍存争议。以SUVmax阈值≥5协助RS诊断时，其敏感性约为96％、特异性为21％、阴性预测值为86％；以SUVmax阈值≥10协助RS诊断时，其敏感性约为71％、特异性为50％、阴性预测值为88％[6]。随着测序技术的发展，单细胞拷贝数变异（sCNA）可识别具有中间态或“动态过渡”特征的细胞群，揭示淋巴组织或骨髓微环境内疾病转化过程中渐进性的基因组紊乱与转录重编程机制[7]。Nadeu等[7]对19例最终发展为RS的CLL患者的纵向肿瘤样本进行多组学联合单细胞DNA测序发现，患者在CLL诊断时已携带RS细胞的基因组、免疫遗传学和转录组特征的微小亚克隆，这些分子驱动亚克隆在转化前休眠时间长达19年。各种检测新技术有效提高RS早期诊断效率，极大提升了早期识别、克隆相关性判定及转化机制研究的能力。检测血浆游离DNA（cfDNA）等无创诊断方式亦具有重要价值，尤其在诊断分型以及疗效监测方面。超低深度全基因组测序（ULP-WGS）技术检测RS患者cfDNA中的sCNA，可能是RS未来研究的重要领域，有助于早期无创性识别RS[8]。

RS分子机制尚不明确。IGHV突变、TP53基因变异、染色体复杂核型、MYC等抑癌基因失活、NOTCH1活化、体细胞核苷酸变异、拷贝数变异事件影响DNA损伤、MAPK与染色质表观遗传学调控、NFκB信号通路异常活化等均是发生RS的高危因素[3]。本文报道病例携带TP53基因（17p13.1）缺失可能是其短期颈部淋巴结进行性肿大的原因。RS体细胞基因突变的非负矩阵分解（NMF）聚类显示5种RS亚型，每种亚型均具有特定的驱动基因，并得到配对转录组分析的支持。其中Tri12/NOTCH1亚型驱动的RS患者具有较低的基因组改变分数和较好的预后，SF3B1/EGR2突变则是首次被识别的RS亚型，TP53突变相关亚型提示预后不良[9]。中南大学湘雅二医院血液内科彭宏凌团队通过对1例RS患者的外周血CLL细胞以及发生转化的淋巴结组织行成对样本分析，应用单细胞RNA测序以及高通量染色体构象捕获技术分别评估细胞类型特异的转录变化以及染色质结构分析，证明CLL与DLBCL细胞在拓扑关联结构域（TADs）存在接触差异，二者之间差异表达基因在cAMP介导信号通路显著富集，表明染色质重组和cAMP信号通路的改变可驱动RS[10]。Nadeu等[7]研究表明，RS细胞发生代谢重编程，使氧化磷酸化（OXPHOS）高表达，导致常规耗氧量（OCR）是CLL细胞的3.5倍，电子传递链容量提升5倍。OXPHOS-high表型依赖线粒体呼吸链亢进，可实现ATP高效供能，进而驱动RS。

目前RS缺乏标准治疗方案，预后不良[11]。大多数RS与既往CLL存在克隆相关性，随着检测技术的发展，已经明确存在与CLL克隆无关的RS。其中，CLL克隆相关的RS患者预后［总生存（OS）期约14.2个月］较非相关RS患者预后（OS期约62.5个月）更差[12]。用于治疗DLBCL的标准及强化免疫化疗方案，以及针对CLL新型靶向药物（BTKi和BCL2抑制剂），对RS患者均未见明显效果[13]。造血干细胞移植被用于RS的缓解后治疗及挽救性治疗，但效果欠佳，auto-HSCT 3年复发率、无进展生存（PFS）率和OS率分别为37％、48％和57％，而allo-HSCT的3年复发率、PFS率和OS率分别为30％、43％和52％[14]。allo-HSCT仅可使少数对诱导治疗有反应的RS患者实现长期缓解[15]。近年来，CAR-T细胞治疗在恶性血液病治疗中迅速发展。TRANSCEND CLL 004研究招募了23例复发/难治CLL/SLL患者接受利基迈仑赛（Liso-cel）治疗，其客观缓解率和完全缓解率分别为82％和45％，其中75％患者达到外周血微小残留病（MRD）阴性，65％患者达到骨髓MRD阴性。中位PFS期为18个月，外周血和（或）骨髓中实现MRD阴性的患者生存期显著延长[16]。CAR-T细胞联合伊布替尼等BTKi治疗复发/难治CLL效果显著，可促进CAR-T细胞增殖并减轻细胞因子释放综合征[17]。一项纳入9例RS患者的研究显示，抗PD-1单抗（帕博利珠单抗）使1例患者疾病完全缓解、3例患者疾病部分缓解，总体中位生存时间为10.7个月（其中3例患者之前未接受过针对RS的靶向治疗）[18]。CAR-T细胞治疗联合免疫检查点抑制剂可有效改善T淋巴细胞耗竭，初步显示出对复发/难治DLBCL较高应答率[19]。此外，对于复发/难治DLBCL患者而言，auto-HSCT联合CAR-T细胞治疗方案比单纯CAR-T细胞治疗的进展率更低、生存率更高[20]。对于RS患者而言，CAR-T细胞治疗及多种方案联用有望成为新的治疗方向。

本例创新性采用抗CD19 CAR-T细胞桥接allo-HSCT治疗策略，使RS患者获得完全缓解。本研究成果为RS患者提供了一种有前景的治疗思路。然而，本研究作为一项单病例报告，其结果存在一定的局限性：个案的成功可能受到患者特定生物学特征、对前期治疗的特殊反应等多种因素影响，其疗效和安全性尚需在前瞻性、多中心的临床试验以及更大规模的患者群体中验证。
